# Laser visual guidance versus two-dimensional vision in laparoscopy: a randomized trial

**DOI:** 10.1007/s00464-016-4937-3

**Published:** 2016-06-17

**Authors:** Stine Maya Dreier Sørensen, Oria Mahmood, Lars Konge, Ebbe Thinggaard, Flemming Bjerrum

**Affiliations:** 1 Copenhagen Academy for Medical Education and Simulation, University of Copenhagen, Blegdamsvej 9, 2100 Copenhagen, Capital Region of Denmark Denmark; 2 University of Copenhagen, Copenhagen, Denmark; 3 Department of Surgery, Roskilde and Koege Hospital, Koege, Denmark

**Keywords:** Lasers, Laparoscopy, Surgical skills, Three-dimensional vision, Simulation training

## Abstract

**Background:**

During laparoscopy, the surgeon’s loss of depth perception and spatial orientation is problematic. Laser visual guidance (LVG) is an innovative technology that improves depth perception to enhance the visual field. In this trial, we examined the effect of LVG on surgical novices’ motor skills, quality of task performance, and cognitive workload.

**Methods:**

We designed a randomized controlled trial following the CONSORT statement. Thirty-two surgical novices completed the Training and Assessment of Basic Laparoscopic Techniques (TABLT) test. The first attempt allowed participants to familiarize themselves with the exercises. We then randomized the participants, and they completed a test session using either LVG or conventional two-dimensional vision.

**Results:**

We found no significant difference between using the LVG tool and conventional 2D vision; however, both the mean completion time and movements used were less in the LVG group: Mean time used in the LVG group was 1288 s (95 % CI 1188–1388) versus 1354 s (95 % CI 1190–1518) (*p* = 0.45); mean angular path length used in the LVG group was 24,049° (95 % CI 20,761–27,336) versus 26,014° (95 % CI 22,059–29,970) (*p* = 0.42); mean path length in the LVG group was 4560 cm (95 % CI 3971–5,149 cm) versus 5062 cm (95 % CI 4328–5797), (*p* = 0.26). Moreover, the mean TABLT performance score was higher in the LVG group compared with the 2D group, although not significant: 379 (95 % CI 352–405) versus 338 (95 % CI 288–387) (*p* = 0.14). No significant difference was found between the groups’ cognitive workloads.

**Conclusion:**

We found no significant improvement of laparoscopic motor skills when using LVG, although a tendency toward improved performance was seen. LVG could have the potential to help novice surgeons acquire basic laparoscopic; however, further development of the concept and validation is needed to confirm this.

Laparoscopic surgery has several benefits for patients, but is difficult for novice surgeons to master [1]. The surgeon’s loss of depth perception and spatial orientation are two challenges to acquiring basic laparoscopic skills [2]. One tool for mitigating these challenges is three-dimensional (3D) vision, which enhances the visual field, decreases cognitive load, and reduces the time for novice surgeons to reach proficiency [3–6]. An alternative, low-cost approach is to use laser visual guidance (LVG) technology as a visual aid during laparoscopy.

An advantage of LVG compared with 3D equipment is that there is no need for 3D glasses, special scopes, or screens. Furthermore, some 3D equipment has previously been shown to cause side effects such as nausea, headache, and dizziness [7–9] which potentially cloud be avoided with the use of LVG.

Lasers have become increasingly popular in a variety of medical fields during recent decades, and their use is rapidly expanding [10–12]. However, to our knowledge, the use of lasers as visual aids in laparoscopy had never been tested. LVG in laparoscopy improves depth perception by projecting a liner grid of laser dots from the instrument tip, and development of the technology is based on the same principles used in graphical design and design of 3D objects, in which grids increase depth perception [13].

The laser grid projected on tissue creates an illusion of depth, which potentially could reduce cognitive load and procedure time compared with conventional two-dimensional (2D) vision. Moreover, LVG may result in more accurate performance (fewer surgical errors), similar to the improved accuracy novice surgeons experience when using 3D vision equipment [2, 3, 7].

The objectives of this trial were to examine whether LVG could reduce completion time and total movements, while improving the quality of tasks performed in a validated laparoscopic training program for surgical novices. Moreover, we compared the subjective cognitive workload of surgeons using LVG with those using 2D vision.

## Materials and methods

### Design

We designed a single-center, randomized superiority trial following the CONSORT statement. The trial received an exemption for ethical approval by the Regional Committee on Biomedical Research Ethics (FSP-15,000,097) and was registered at Clinicaltrials.gov (NCT02407483).

### Trial intervention

The Training and Assessment of Basic Laparoscopic Techniques (TABLT) test [14] consists of five different exercises: midi-bead transfer, cutting, sharp dissection, blunt dissection, and cyst removal on a box trainer [14]. All participants were required to read the standardized description of the tasks and definition of errors for the TABLT test before performing the test. The participants received feedback during the first attempt and could freely ask questions of the investigators (SS or OM). After the first attempt, we randomized participants to complete the TABLT test again, using either LVG or conventional 2D vision. During the second test, participants were not allowed to ask questions and did not receive feedback. After the second attempt, participants completed the workload questionnaire, National Aeronautics and Space Administration Task Load Index (NASA-TLX) [15]. All data collection was done at the Simulation Center at Rigshospitalet, Copenhagen [16].

### Outcome measures

The primary outcome was motor skills performance parameters during the TABLT test session including, the total task time (minutes), the total angular path (cumulative angular degrees for both the right and left instrument, measured in degrees), and the total path length (cumulative distance tip movement for both the right and left instrument, measured in centimeters).

The secondary outcome was the TABLT performance score, a combination of time and errors for the five exercises. The score could range between 0 and 708 [14]. An external rater performed video ratings (FB) and calculated performance scores.

The exploratory outcome was a subjective workload questionnaire (NASA-TLX), which consists of six dimensions: mental demand, physical demand, temporal demand, performance success, effort, and frustration [15, 17].

### Participants

The participants included in the trial were surgical novices (medical students). We sent them an email invitation to participate, and each received both written and verbal information before giving their written consent to participate. The inclusion and exclusion criteria used were as follows:

- *Inclusion criteria* (1) medical students enrolled at The Faculty of Health Science, University of Copenhagen, who (2) provided informed consent.
- *Exclusion criteria* (1) participation in prior trials involving laparoscopic training; (2) experience with laparoscopy surgery (having performed any laparoscopic procedure); (3) failure to provide informed consent; or (4) inability to speak Danish on a conversational level.

All participants were assigned a unique trial identification number before randomization.

### Sample size calculation

We performed an a priori sample size calculation based on the results from a pilot study. We expected that the intervention group would use 1300 s to complete the five exercises in the TABLT, test compared with 1600 s for the control group, and we assumed a standard deviation of 300 for both groups. With a two-sided significance level of 0.05 and a power of 0.80, the minimum sample size required was 32 participants, 16 in each group.

### Randomization

To assigned participants to the two groups, we used a web-based system from Sealed Envelope (www.sealedenvelope.com, London, United Kingdom), employing a 1:1 randomization model [18]. The computer-based random allocation sequence used block sizes of two and four and was kept concealed from the investigators throughout the trial. Participants were stratified according to sex (man/woman), as previous trials have found sex to be a predictor of initial laparoscopic simulator performance [19]. The investigators (SS or OM) randomized the participants, who were then allocated to either the intervention or control group after they were familiarized with the test. The participants performed the TABLT test with LVG or with 2D vision immediately after randomization.

### Materials and equipment

**Fig. 1:**
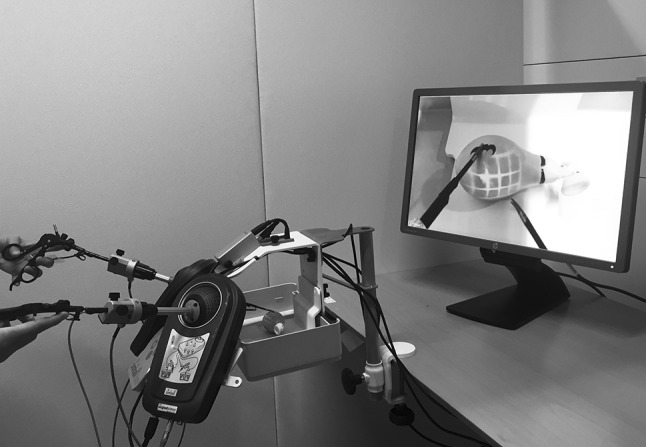
The simulation test setup

**Fig. 2:**
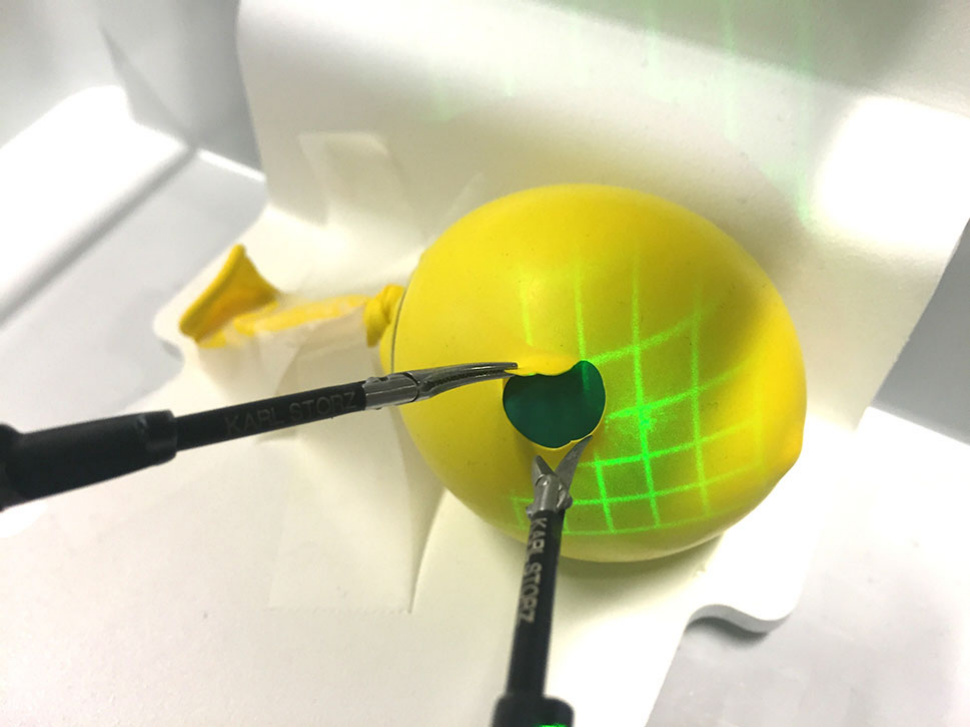
Laser visual guidance (LVG) during the cyst dissection task

Two simulators (Simball Box, G-coder systems, Västra Frölunda, Sweden) were used, and the five exercises were divided as following: *simulator one*—midi-bead transfer and blunt dissection using two 3-mm graspers (Karl Storz, Tuttlingen, Germany); *simulator two*—sharp dissection, cutting and cyst removal using a 3-mm grasper and a 3-mm scissors (Karl Storz, Tuttlingen, Germany). Both simulators were connected to a computer, which stored all data automatically. The simulators consisted of a physical user interface with adapters where the laparoscopic instruments could be inserted (see Fig. 1). We used Simball Box software (Simball Box, version 2015, G-coder systems, Västra Frölunda, Sweden) to record the instrument tip movement (angular path length and path length) and the task completion time. The laser prototype (3Dintegrated ApS, Copenhagen, Denmark) was incorporated into the laparoscopic instruments on the right side of both simulators. The laser color was green and consisted of a grid of 6-by-6 squares. The smallest square laser grid was 3 cm by 3 cm, when the tip of the instrument touched the task (e.g., when the laparoscopic instrument touched the balloon in the cyst removal exercise). When moving away from the task, the grid became larger hereby creating an illusion of depth (see Fig. 2).

### Statistical analyses

We used statistical software (SPSS, version 22.0, IBM, Armonk, NY, USA) to analyze the data. We tested equality of variances using Levene’s test, and we used either Student’s *t* test or Welsh *t* test accordingly for comparisons of primary, secondary, and explorative outcomes. *p* values of less than 0.05 were considered statically significant.

## Results

**Fig. 3:**
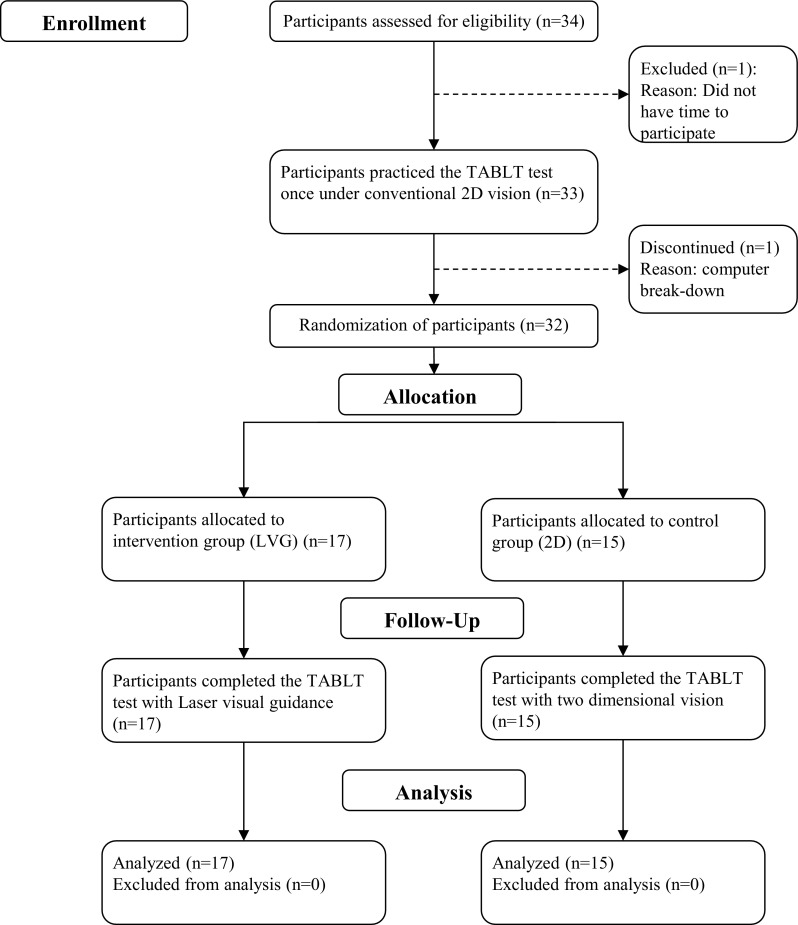
Trial flowchart in accordance with the CONSORT statement

Thirty-two out of the 34 included participants completed both the laparoscopic training and the TABLT test, (Fig. 3). Two participants dropped out of the trial: The first did not have enough time to participate in the trial and withdrew before randomization, and the second was excluded from the trial during the first attempt because of technical problems with the simulator, which could not be resolved. The participants’ characteristics of age, sex, and dexterity were distributed equally between the two groups (Table 1).

**Table 1:** Participant baseline characteristics

	Intervention group (LVG) (*n* = 17)	Control group (2D) (*n* = 15)
Sex, number of men/women	12/5	10/5
Age in years, mean/range	24/20–32	25/21–36
Dexterity, number of right-/left-handed	17/0	13/2

The outcome measures of the LVG had smaller variances than the control group, but this finding was statistically significant only for the TABLT performance score (*p* = 0.03).

**Fig. 4:**
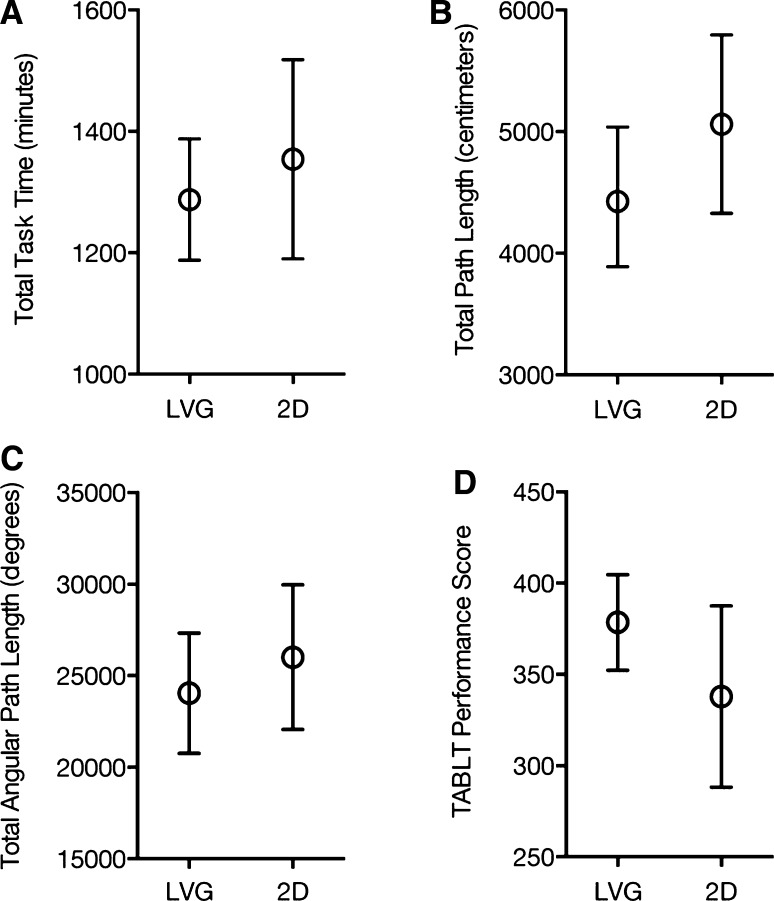
Error plots (with 95 % CI) for the intervention group (LVG) and the control group (2D) of the outcomes: **A** total task time (*p* = 0.45), **B** total path length (*p* = 0.26), **C** total angular path length (*p* = 0.42) used to complete the TABLT test and **D** the TABLT performance score (*p* = 0.14)

We found no significant differences between the LVG tool and 2D vision in regard to motor skill performance parameters (Fig. 4). The mean time to completion in the LVG group was 1288 s (95 % CI 1188–1388) versus 1354 s (95 % CI 1190–1518) in the 2D group (*p* = 0.45). The mean angular path length used in the LVG group was 24,049° (95 % CI 20,761–27,336) versus 26,014° (95 % CI 22,059–29,970) in the 2D group (*p* = 0.42). The mean path length used in the LVG group was 4560 cm (95 % CI 3971–5,149 cm) versus 5062 cm (95 % CI 4328–5797 cm) in the 2D group (*p* = 0.26).

The TABLT performance score, which combined errors and time for all five exercises, showed a tendency toward improvement when using LVG. The mean score was 379 (95 % CI 352–405) versus 338 (95 % CI 288–387) in the 2D group, but the difference was not statistically significant (*p* = 0.14), (Fig. 4).

To examine whether LVG reduced the subjective workload during the TABLT test, we used the NASA-TLX, consisting of six dimensions of workload. The questionnaire revealed no significant difference between the LVG group and the 2D group (Table 2).

**Table 2:** Comparison of subjective workload scores for novices using the National Aeronautics and Space Administration Task Load Index (NASA-TLX)

	Intervention group (LVG)	Control group (2D)	*p* value
Mental demand	64 (23)	55 (22)	0.23
Physical demand	57 (26)	56 (19)	0.86
Temporal demand	50 (18)	46 (24)	0.58
Performance success	32 (15)	33 (17)	0.83
Effort	74 (16)	74 (15)	0.99
Frustration	43 (26)	39 (27)	0.70

Mean (SD) for the workload scale variables (0–100), after completion of the TABLT test

## Discussion

We found no significant difference between the LVG tool and conventional 2D vision. Nevertheless, we found that there was a tendency toward improvements in the LVG group as they required less time and fewer movements to complete the TABLT test and appeared to have a superior quality of tasks performance. We found no significant difference in mental workload using a subjective workload questionnaire.

The results and experiences from our trial represent the first LVG prototype used for laparoscopy. Because of the novelty of this equipment, we cannot compare our results to those in other trials. However, the idea of a device that can improve depth perception during laparoscopy is not new. Several trials in the last two decades have examined the effect of 3D vision in laparoscopy [8, 9, 20–30]. Recent studies have demonstrated positive effects on task performance, such as reduced time to completion and improved precision [3, 5, 21, 31]. Our findings show the same tendency, and we believe that LVG has the potential to help novice surgeons acquire basic laparoscopic skills in the future, although further developments are needed. We base this assumption on the Wickens Multiple Resource Theory (MRT) [32–34], which postulates that humans have multiple cognitive resources that can be accessed simultaneously. However, as there is a limited processing capacity in a single cognitive resource, too much information can negatively affect the performance of a task, resulting in longer time to completion and a higher number of errors. The problem with a limited processing capacity occurs when an individual performs two or more tasks that require a single resource, such as having to focus on performing a procedure and to look at the operating field, while at the same time compensating for the loss of depth perception. LVG is a tool that reintroduces binocular depth cues, and therefore will theoretically make movements faster and more precise.

The test setup could explain why we could not find a significant improvement. In our simulated setup, we used a simple box trainer with a flat platform and a static background. This setup could have made it difficult for the participants to appreciate the LVG depth clues. Instead, if the test environment had been similar to an actual operating environment, the utility of LVG may have been more pronounced. Moreover, development of an on/off button to allow the novice surgeon to choose when to use the LVG grid could improve the LVG tool’s utility and the surgeon’s performance. The laser light is especially useful for guidance toward a target, but when placing the instrument on a target area and when working in this area, the laser light may cause more disturbance than help. An additional limitation is that the laser light was only available on the right instrument and left-handed participants could have had difficulties in proper use of the laser. These adjustments set the stage for future trials, which will aim to demonstrate the effectiveness of LVG.

Currently, effective surgical training programs are a high priority because of the restricted resident duty hours. Restricted duty hours make it difficult for trainees to reach competency in laparoscopic surgery during specialty training [35], and therefore, examining different ways of optimizing the surgical training programs is highly relevant. The use of LVG could accelerate the initial learning curve for surgical novices, thereby making surgical training programs more effective.

Moreover, superior performance is the trademark of expertise, but more consistent performance is also important, according to the Fitts and Posner three-stage model of acquiring expertise [36]. The outcome measures of the LVG group had smaller variances, which indicate more consistent performance. Visual guidance could potentially assist the most insecure novices and help to avoid substandard operations. However, we acknowledge that our findings were significant only regarding the TABLT score and the hypothesis of elimination of very inferior performances must be tested in a separate study.

We recommend that future research on the LVG prototype should be performed in a more realistic testing environment, similar to that of real laparoscopic operations. Future research on LVG should aim to clarify the utility of LVG during actual operations and in laparoscopic training programs.

In conclusion, we did not find a significant improvement in laparoscopic motor skills when using LVG. Adjustments of the LVG prototype and further studies are necessary to demonstrate the full potential of this novel technology.
